# Patterns of failure after immunotherapy with checkpoint inhibitors predict durable progression-free survival after local therapy for metastatic melanoma

**DOI:** 10.1186/s40425-019-0672-3

**Published:** 2019-07-24

**Authors:** Nicholas D. Klemen, Melinda Wang, Paul L. Feingold, Kirsten Cooper, Sabrina N. Pavri, Dale Han, Frank C. Detterbeck, Daniel J. Boffa, Sajid A. Khan, Kelly Olino, James Clune, Stephan Ariyan, Ronald R. Salem, Sarah A. Weiss, Harriet M. Kluger, Mario Sznol, Charles Cha

**Affiliations:** 1 Section of Surgical Oncology, Yale School of Medicine, 20 Park Street, New Haven, CT 06519 USA; 2 Department of Radiology, Yale School of Medicine, New Haven, CT USA; 3 Orlando Health Aesthetic and Reconstructive Surgery Institute, Orlando, FL USA; 4 Division of Surgical Oncology, Oregon Health and Science University, Portland, OR USA; 5 Section of Thoracic Surgery, Yale School of Medicine, New Haven, CT USA; 6 Section of Plastic and Reconstructive Surgery, Yale School of Medicine, New Haven, CT USA; 7 Section of Medical Oncology, Yale School of Medicine, New Haven, CT USA

**Keywords:** Melanoma, Immunotherapy, Metastasectomy, Local therapy, Checkpoint inhibitors, Checkpoint blockade, Pattern-of-failure

## Abstract

**Background:**

Checkpoint inhibitors (CPI) have revolutionized the treatment of metastatic melanoma, but most patients treated with CPI eventually develop progressive disease. Local therapy including surgery, ablation or stereotactic body radiotherapy (SBRT) may be useful to manage limited progression, but criteria for patient selection have not been established. Previous work has suggested progression-free survival (PFS) after local therapy is associated with patterns of immunotherapy failure, but this has not been studied in patients treated with CPI.

**Methods:**

We analyzed clinical data from patients with metastatic melanoma who were treated with antibodies against CTLA-4, PD-1 or PD-L1, either as single-agent or combination therapy, and identified those who had disease progression in 1 to 3 sites managed with local therapy. Patterns of CPI failure were designated by independent radiological review as growth of established metastases or appearance of new metastases. Local therapy for diagnosis, palliation or CNS metastases was excluded.

**Results:**

Four hundred twenty-eight patients with metastatic melanoma received treatment with CPI from 2007 to 2018. Seventy-seven have ongoing complete responses while 69 died within 6 months of starting CPI; of the remaining 282 patients, 52 (18%) were treated with local therapy meeting our inclusion criteria. Local therapy to achieve no evidence of disease (NED) was associated with three-year progression-free survival (PFS) of 31% and five-year disease-specific survival (DSS) of 60%. Stratified by patterns of failure, patients with progression in established tumors had three-year PFS of 70%, while those with new metastases had three-year PFS of 6% (*P* = 0.001). Five-year DSS after local therapy was 93% versus 31%, respectively (*P* = 0.046).

**Conclusions:**

Local therapy for oligoprogression after CPI can result in durable PFS in selected patients. We observed that patterns of failure seen during or after CPI treatment are strongly associated with PFS after local therapy, and may represent a useful criterion for patient selection. This experience suggests there may be an increased role for local therapy in patients being treated with immunotherapy.

## Background

Advances in cancer immunotherapy have dramatically changed the treatment landscape for patients with metastatic melanoma. Durable complete responses have been recorded following high-dose bolus interleukin-2 (IL-2) [1–3], adoptive cell transfer (ACT) [4, 5] and checkpoint inhibition (CPI) using antibodies against CTLA-4, PD-1 or PD-L1 [6–10]. However, a majority of patients treated with immunotherapy are either primary non-responders or eventually develop immunorefractory progressive disease and require additional therapy.

In contrast to up-front metastasectomy, which can be curative in selected patients with oligometastatic disease [11, 12], there are few data describing the use of local therapy (henceforth defined as metastasectomy, ablation or stereotactic body radiotherapy) to manage extra-cranial oligoprogression after immunotherapy. Small series showed that surgery for highly selected patients who progressed after IL-2 or CPI could achieve long PFS [13, 14] and early reports of the Memorial Sloan Kettering Cancer Center (MSKCC) experience quote 60% survival after surgery for a solitary site of progression [15].

Retrospective pattern-of-failure data in patients with non-small cell lung cancer (NSCLC) led to the initiation of two prospective randomized trials of local therapy plus systemic therapy versus local therapy alone [16, 17]. Both trials demonstrated that improved PFS could be achieved by treating established tumors present at baseline, but in neither study was durable PFS achieved by local therapy. However, in a series of 26 patients with metastatic melanoma treated with ACT, investigators in the Surgery Branch, NCI showed that metastasectomy could achieve durable PFS [18], and furthermore showed that patterns of failure after ACT strongly associated with the duration of PFS and OS after surgery. In patients with progression in established tumors present at baseline, resection achieved median PFS of 46 months, and actuarial five-year survival was 73%. In contrast, patients who had resection of new metastases, which had developed after ACT, had median PFS of 3 months and five-year survival of 0%.

For the present study, we reviewed our entire institutional experience of 428 patients with metastatic melanoma treated with CPI. We sought to determine when local therapy was used to control oligoprogression of metastatic disease, and whether patterns of failure after CPI were associated with different outcomes after local therapy.

## Methods

### Treatment with checkpoint inhibitors (CPI)

Patients with metastatic melanoma received treatment with antibodies against CTLA-4 (Ipilimumab), PD-1 (Nivolumab, Pembrolizumab) and/or PD-L1 (Atezolizumab). Patients who were only treated with CPI in the adjuvant setting were excluded. Treatment with other immunotherapy agents (Interferon, IL-2 or ACT) before CPI was not an exclusion. Some patients were treated under the auspices of clinical trials which were reported elsewhere [7, 8, 10].

### Local therapy

The Yale Multi-Disciplinary Melanoma Tumor Board evaluated all patients presenting with metastatic disease before treatment with CPI. Up-front local therapy was often rejected because of technical considerations or an insignificant probability of long-term benefit. After definitive treatment with CPI for metastatic disease, patients who developed progression in 1–3 sites that were managed with local therapy were included for analysis. We excluded patients who required local therapy within 6 months of starting CPI; this cutoff served as a biologic test for the efficacy of immunotherapy, and a similar threshold was used in a prior study [18]. The exact indications for local therapy in each patient were retrospectively collected. All surgical specimens had viable melanoma by pathologic analysis. We excluded procedures performed solely for diagnosis, research, palliation or T cell harvest. Patients subjected to multiple local therapies were only included once and were analyzed based on the first procedure. Local therapy for 2–3 lesions could be performed in a staged manner if the intent was documented before the first procedure. Incomplete (R2) resections and failed staged procedures were included and categorized to reflect intent-to-treat. We did not study local therapy applied to the CNS, but patients staged M1d could be included if they did not have active brain metastases at the time of local therapy.

### Treatment strategy

The goal of local therapy was either to achieve no evidence of disease (NED) status or non-progressive residual disease (NPRD) status by eliminating 1–3 sites of progressive disease. No patient could have more than 3 sites of progression, but NPRD patients could be left with any number of stable or regressing tumors, as demonstrated by at least two serial imaging evaluations. Categorization as NED or NPRD reflected intention-to-treat.

### Designation of patterns of failure

A radiologist who was blinded to the study hypothesis and design evaluated all progressing tumors and indicated when they were first apparent on cross-sectional imaging (CT, MRI) or PET scan. Progressing tumors evident before the first cycle of CPI were classified as “established”; those appearing after were classified as “new.” All progressing tumors had to be present before CPI in order to be categorized as “established.” Physical exam findings documented in clinical notes were used for extremity lesions that were not adequately imaged.

### Statistical analysis

The primary endpoint was progression-free survival (PFS); secondary endpoints were disease-specific survival (DSS), morbidity related to local therapy, 90-day mortality and technical failures (R2 resections, persistent enhancement of ablated or irradiated lesions, or incomplete staged procedures). Time-to-event curves start with the first dose of CPI and each event indicates local therapy for one patient. Survival is shown using the Kaplan-Meier method, starting with the date of local therapy. Univariate comparisons used the log-rank test. Continuous variables are shown as median and range, discrete variables as frequency. Demographic data were compared by Kruskal-Wallis (age) or Chi-square methods.

## Results

**Fig. 1:**
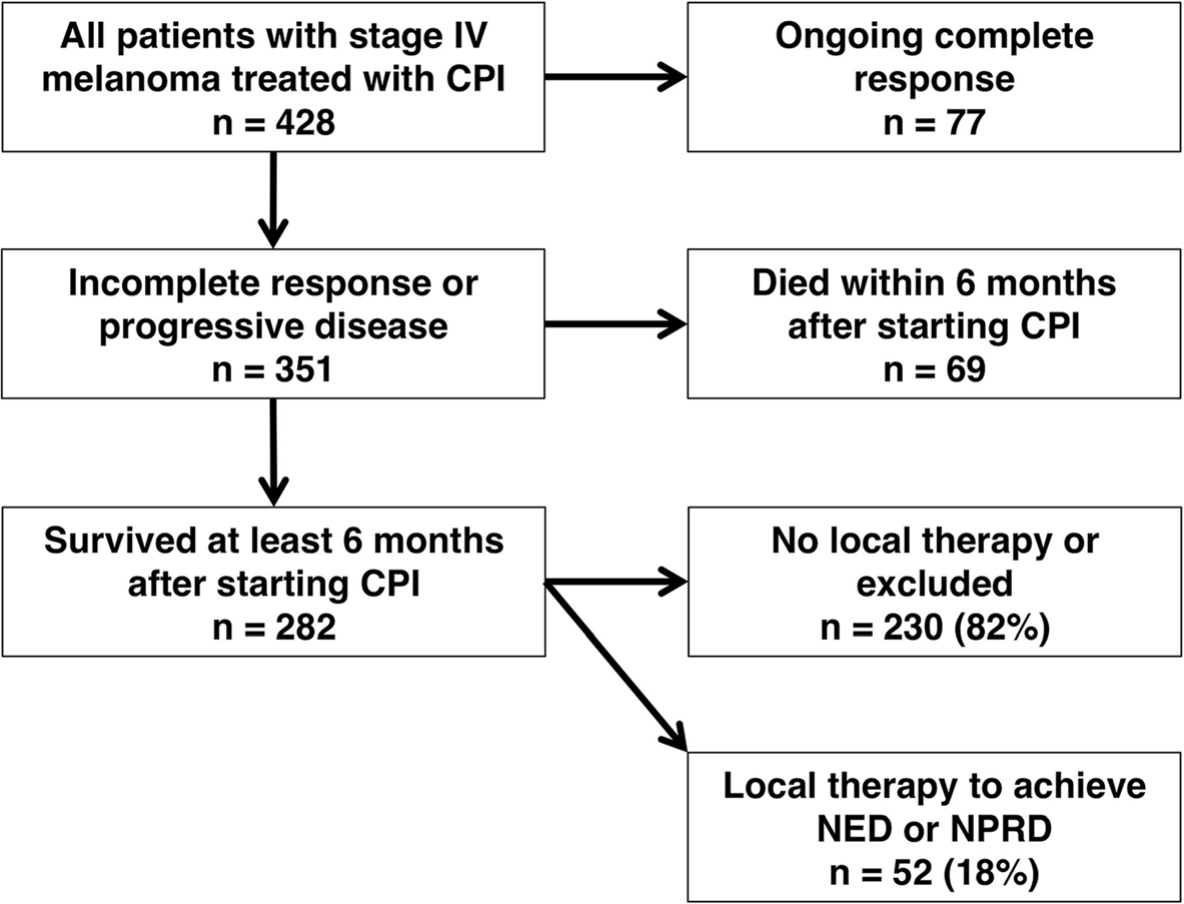
Consort diagram for patient selection. Abbreviations: *CPI* = checkpoint inhibition; *NED* = no evidence of disease; *NPRD* = non-progressive residual disease

Four hundred twenty-eight patients with metastatic melanoma were treated with CPI from 2007 to 2018, 187 (44%) under the auspices of a clinical trial and 241 (56%) under standard of care (Fig. 1). With a median follow-up of 45 months, median overall survival for the entire population (*n* = 428) from the first cycle of CPI was 34 months and actuarial five-year survival was 41%. These data are similar to that reported in the CheckMate 067 trial, but in our series 87 patients (20%) had brain metastases and 35 (8%) had ocular melanomas, both of which were excluded from the Checkmate 067 study [19]. Furthermore, some patients in our experience only received treatment with anti-CTLA-4, and were never treated with anti-PD-1 or anti-PD-L1.

Seventy-seven of the 428 patients (18%) had ongoing complete responses at most recent follow-up. These patients were less likely to have sun-shielded primary tumors (*P* < 0.01) or be staged M1d (*P* < 0.01) (Table 1). Sun-shielded melanomas, including ocular, mucosal and acral lentiginous melanomas, are considered less responsive to CPI and have worse outcomes, potentially due to a lower burden of neoantigens [21–23]. The 77 patients with ongoing complete responses were also more likely to have been treated with combination CTLA-4 and PD-1 blockade (*P* < 0.01). Meanwhile, 69 of 428 patients (16%) died within 6 months of starting CPI; these patients had advanced stages and were less likely to be treated with combination CPI **(**Table 1**)**.

**Table 1:** Demographic data, stage, primary tumor histology and type of first CPI treatment

	All patients (*n* = 428)	Ongoing CR (*n* = 77)	Died < 6 months (*n* = 69)	No local therapy** (*n* = 230)	NED or NPRD (*n* = 52)
Median age (IQR)	65 (55 – 74)	68 (55 – 75)	65 (58 – 74)	64 (55 – 74)	61 (54 – 70)
Gender	63% male	68% male	67% male	61% male	62% male
Stage before CPI Stage M1a (%) Stage M1b (%) Stage M1c (%) Stage M1d (%)	99 (23%) 90 (21%) 152 (36%) 87 (20%)	22 (29%) 21 (27%) 31 (40%) 3 (4%)	11 (16%) 8 (12%) 36 (52%) 14 (20%)	55 (24%) 53 (23%) 69 (30%) 53 (23%)	11 (21%) 8 (15%) 16 (31%) 17 (33%)
Sun-shielded*	93 (22%)	5 (6%)	19 (28%)	54 (23%)	15 (29%)
1^st^ Therapy (%) CTLA4 PD1 or PDL1 CTLA4 + PD1	124 (29%) 129 (30%) 175 (41%)	11 (14%) 23 (30%) 43 (56%)	28 (41%) 23 (33%) 18 (26%)	69 (30%) 71 (31%) 90 (39%)	16 (31%) 12 (23%) 24 (46%)

Abbreviations: *CR = complete response; IQR = interquartile range; NED = no evidence of disease; NPRD = non-progressive residual disease*

Stage M1a = distant metastases to skin or lymph nodes. Stage M1b = metastases to lung with or without M1a metastases. Stage M1c = metastases to visceral sites with or without M1a or M1b. Stage M1d = metastases to CNS with or without extra-cranial metastasis [20]

*Sun-shielded melanomas include mucosal, ocular, perineal, and acral lentiginous

**Some patients had procedures that did not meet inclusion criteria

There were thus 282 patients who survived at least 6 months and had progressive disease, incomplete responses, or progression in one or more lesions after a partial or complete response. Of this group, 52 patients (18%) had local therapy for extra-cranial oligoprogression and represent the focal point of this study. The remaining 230 patients either had no local therapy or had procedures that did not meet our inclusion criteria. The 52 patients were similar to the 230 with respect to median age (61 years vs. 64 years), gender distribution (62% male vs. 61% male, *P* = 0.96), percentage with a sun-shielded primary tumor (29% vs. 23%, *P* = 0.40) and M1d stage (33% vs. 23%, *P* = 0.25) **(**Table 1**)**.

### Timing and approach of local therapy

**Fig. 2:**
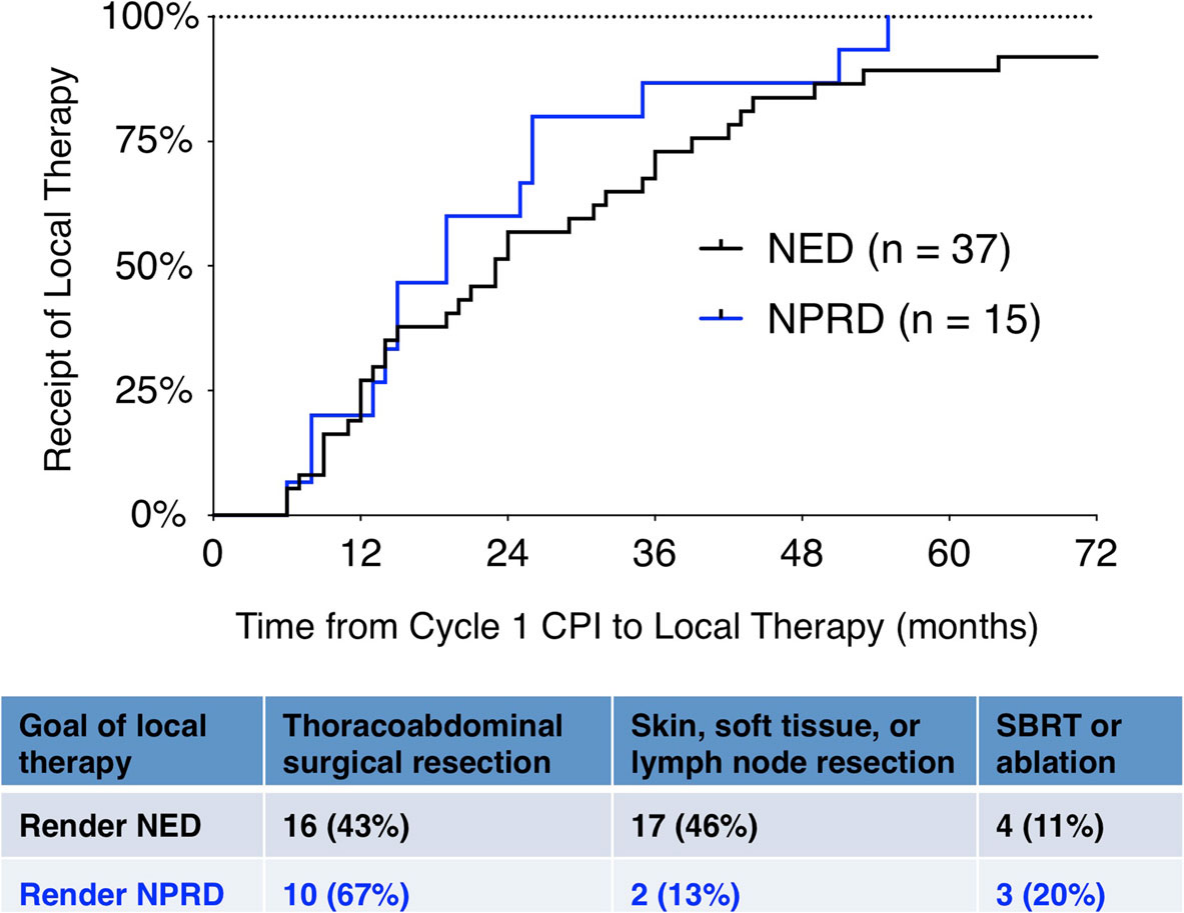
Time to local therapy for the patients rendered NED or NPRD. Time zero corresponds to the date of the first cycle of CPI, with each event corresponding to the date of local therapy for one patient. Three patients left NED had procedures after 72 months, which are not shown. The type of local therapy is listed below

The goal of local therapy was to achieve NED in 37 of 52 patients and NPRD in 15 of 52 (Fig. 2). The median time from the first cycle of CPI to local therapy was 23 months for NED patients (interquartile range, IQR, 11–36) and 19 months for NPRD patients (IQR 14–25; *P* = 0.27). Thus, most procedures were performed in the second or third year after CPI. Half of all procedures were major thoracoabdominal surgical operations (16 of 37 NED, 10 of 15 NPRD; *P* = 0.08). Of the remaining procedures, 19 were operations for tumors in the skin, soft tissues or lymph nodes, while 7 patients had SBRT or ablation for visceral metastases. After local therapy, the treatment team planned to continue CPI in 6 of 37 NED patients and 10 of 15 NPRD patients (*P* < 0.01).

### Complications

Post-operative morbidity occurred in 8 patients (15%); 3 patients had a wound infection and one patient each had a urinary tract infection, ileus, acute kidney injury, bowel obstruction and atrial fibrillation. Complications were minor or transient, with the exception of the bowel obstruction, which required operative adhesiolysis. There were no deaths within 90 days of local therapy. Technical failures occurred in 5 patients (9%); three patients had R2 resections and two had incomplete staged procedures. All 5 relapsed at short follow-up and are actively being treated or have expired.

### Outcomes after local therapy

**Fig. 3:**
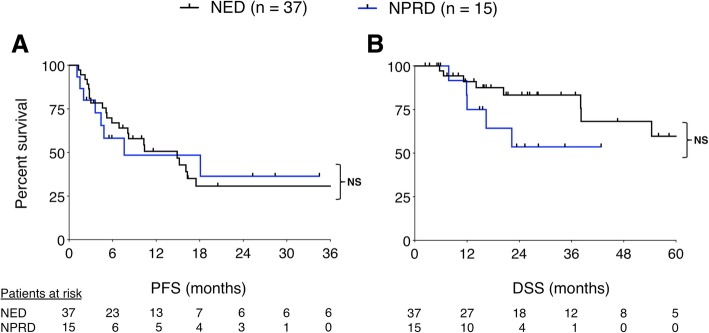
Kaplan Meier curves showing progression-free (**a**) and disease-specific (**b**) survival, calculated from the date of local therapy. PFS curves show 3 years while DSS curves show 5 years. *NS* = not significant; *P* > 0.05 by log-rank test

**Fig. 4:**
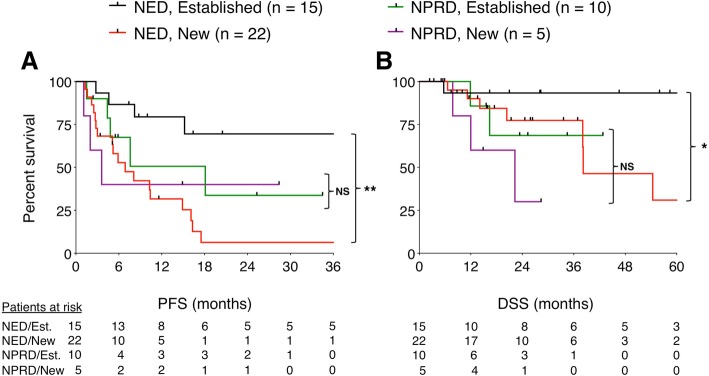
Kaplan Meier curves showing progression-free (**a**) and disease-specific (**b**) survival, stratified by patterns of failure. PFS curves show 3 years while DSS curves show 5 years. * Denotes *P* < 0.05 by log-rank test. ** Denotes *P* < 0.01 by log-rank test

First, we compared outcomes based on the treatment strategy. Median PFS after local therapy to achieve NED versus NPRD was 15 months and 8 months respectively (*P* = 0.91), and median DSS was not reached in either group (*P* = 0.12) (Fig. 3). Next, we stratified the entire cohort by patterns of failure. There were 25 patients who progressed in established metastases and 27 patients who progressed with new metastases; median PFS after local therapy was 40 months versus 7 months, respectively (*P* < 0.01). When we stratified NED and NPRD patients by patterns of failure, we found that patients with established sites of progression who were rendered NED (*n* = 15) had three-year PFS of 70% and five-year DSS of 93% (Fig. 4). In contrast, patients with new metastases who were rendered NED (*n* = 22) had three-year PFS of 6% (*P* = 0.001) and five-year DSS of 31% (*P* = 0.046). Of the NPRD patients, 10 had progressed in established tumors and 5 in new tumors; median PFS after local therapy was 18 months and 4 months, respectively (*P* = 0.53) and median DSS was not reached versus 22 months (*P* = 0.26).

**Fig. 5:**
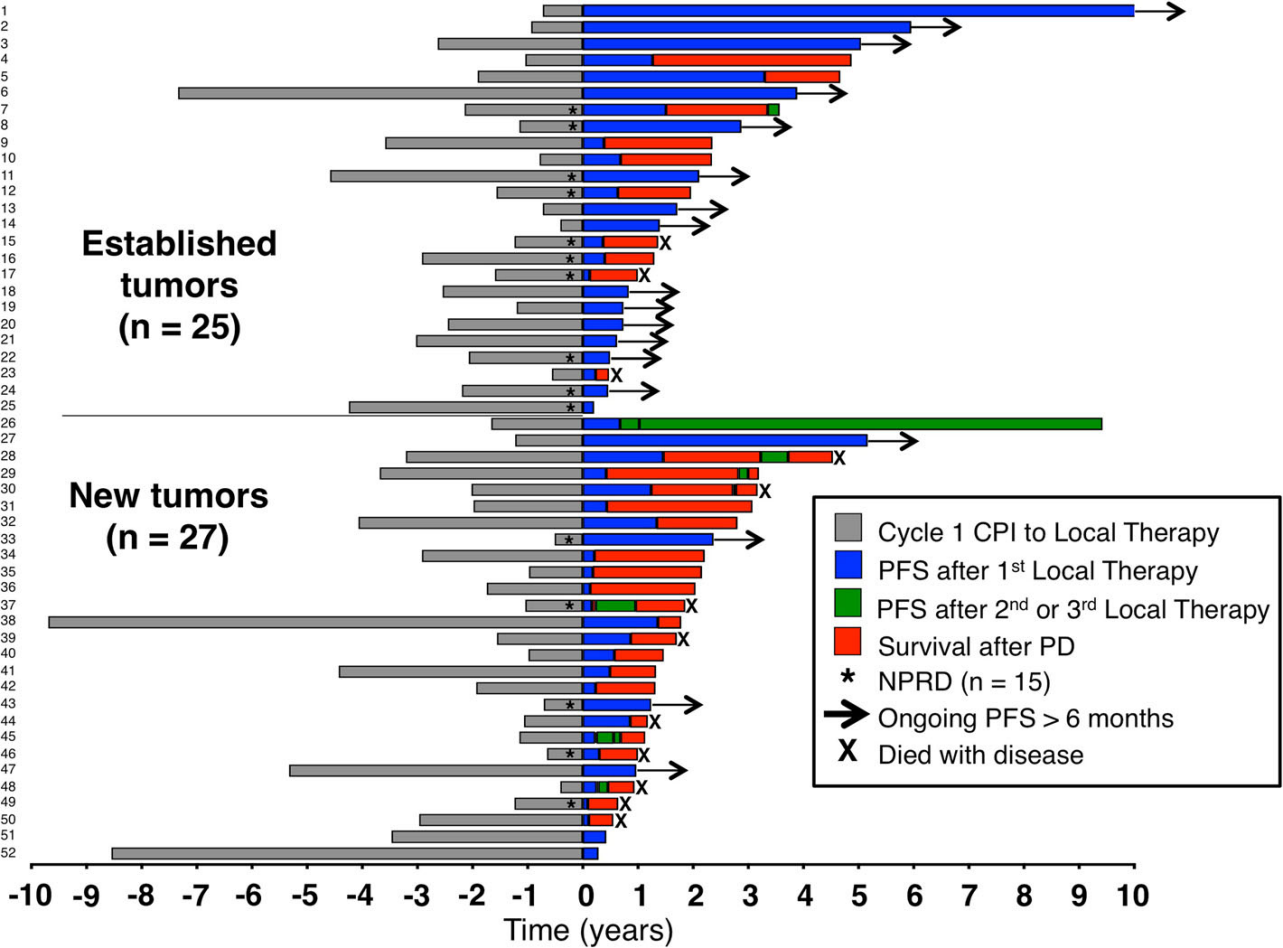
Global treatment landscape of patients rendered NED or NPRD. The date of local therapy is represented as year zero. Patients rendered NPRD are denoted with an asterisk (*). The top 25 patients had progression in established tumors while the bottom 27 had progression with new tumors

In order to perform a global comparison of the patients who had local therapy for established or new metastases, we plotted the treatment landscape of each individual patient (Fig. 5). There was no significant difference in the time from CPI to local therapy, which occurred at a median of 23 months in the established group and 21 months in the new metastasis group (*P* = 0.44). Among the 25 established progression patients, there were 9 who were staged M1c and 10 staged M1d; of the 27 patients with a new site of progression, 6 were staged M1c and 8 were M1d. After local therapy, 31 patients progressed again and 8 (26%) were treated with another procedure that would have met our inclusion criteria (patients 7, 26, 28, 29, 30, 37, 45, 48). One patient had PFS lasting almost 10 years after having three surgical procedures (patient 26).

Finally, we sought to determine whether other clinical variables might associate with outcomes. We compared patients with a solitary site of progression (*n* = 38) with those who had 2–3 sites of progression (*n* = 14). Local therapy was followed by PFS of 16 months and 4 months, respectively (*P* = 0.11) and median DSS was not reached in either group (*P* = 0.11). Of the 38 patients with solitary site of progression, 19 were in established tumors while 19 were new tumors; their median PFS was 40 months versus 8 months, respectively (*P* = 0.003) and five-year DSS was 91 and 19% (*P* = 0.038).

Eight of 52 patients included in this study had achieved a complete response to CPI before progression in a solitary site of metastasis, which was then treated with local therapy to achieve NED. Seven (88%) progressed by developing a new metastasis while one had progression in an established adrenal metastasis that was thought to have resolved. All 7 of the patients with a new metastasis progressed again after local therapy, and 4 have died. Conversely, the patient who recurred in an established site underwent adrenalectomy and has been progression-free for more than 5 years.

Interestingly, while patients with sun-shielded primary tumors (*n* = 15) had similar PFS as those with sun-exposed cutaneous primary tumors, they had worse DSS (*P* = 0.02). Other variables including M1d stage before CPI, major thoracoabdominal surgical resection, and treatment with monotherapy (i.e., anti-CTLA-4, anti-PD-1 or anti-PD-L1 rather than a combination of anti-CTLA-4 and anti-PD-1) were not associated with worse PFS or DSS.

## Discussion

Immunotherapy with CPI has revolutionized the treatment of metastatic melanoma, although most patients will eventually develop disease progression. Here, we have described a single institutional experience using local therapy for selected patients treated with CPI who developed 1–3 sites of progression outside of the CNS. These procedures were technically successful in over 90% of attempts, complications were consistent in incidence with outcomes expected from the procedures performed, and there were no 90-day mortalities.

Local therapy to achieve NPRD makes sense in the context of recent advances in systemic therapy, which are capable of mediating durable regression of widely metastatic disease [6]. Patients rendered NPRD in this study had demonstrated unequivocal evidence of anti-tumor immune responses before developing oligoprogression in 1–3 sites, which appeared to be escape lesions. Other reports have documented that favorable outcomes after metastasectomy are associated with complete resection of all disease [24]. In this report, it should be emphasized that in NPRD patients all *progressive* disease was eliminated by local therapy; only stable or regressing residual disease was left in situ. It is even possible that some residual disease sites were sterile and no longer had active cancer. Furthermore, NPRD patients were more likely to be continued on CPI therapy after their procedures than those who were rendered NED, which serves to illustrate that the management of these patients was unique. This approach, of using local therapy as an adjuvant to CPI, resulted in PFS that was comparable to the patients who were made NED **(**Fig. 3**)**. Ultimately, more data are needed to determine whether local therapy to achieve NPRD is beneficial for patients. In our opinion it would be ethical to study CPI with or without resection to NPRD in a prospective randomized trial.

In a series of highly selected patients, resection of escape lesions in 20% of patients who had an objective response to IL-2 was curative [13]. In a larger series of patients with progressive disease after ACT, it was shown that long PFS could be achieved by surgery in selected patients and that patterns of failure after ACT were associated with different outcomes after surgery [18]. In this report, we show that patterns of failure after CPI also predict outcomes after local therapy. This is intriguing because, to our knowledge, data from large immunotherapy trials have not shown that patterns of failure carry prognostic weight. For example, in a prospective randomized trial of 101 patients treated with adoptive transfer, 38 patients developed new sites of metastasis while 28 progressed in established sites [4]. Patients with disease progression after ACT had high risk of mortality regardless of how their treatment failed. Furthermore, the significance of a new site of metastasis was downgraded in the immune-related response criteria (irRC), which was derived from CPI response patterns [25]. Whereas in RECIST the appearance of a new site of metastasis automatically denotes progressive disease, in the irRC a new site only contributes to it.

Observations of patients achieving a complete response to immunotherapy support the notion that the disappearance of a metastasis generally implies sterilization. In most patients, an immune-mediated complete response is durable for many years and is potentially curative [5, 6]. When patients do relapse after a complete response, they appear to most frequently do so in new sites of metastasis. For example, a study looking at recurrence patterns after IL-2 showed that relapse after a complete response occurred at a new site of metastasis in 70% of cases [26]. In our series, 7 of the 8 patients (88%) who recurred after a complete response did so in a new site. This implies that in some patients, immune-based treatments can eradicate all clinically evident macrometastases but still fail to clear occult micrometastases, which later progress as new metastases.

While it is likely that the appearance of a new metastasis is a marker of aggressive biology, that explanation does not fully account for why a new site doesn’t appear to carry prognostic weight for all-comers, nor does it explain why patients with other favorable prognostic factors frequently do poorly if they progress in a new site. We hypothesize that new metastases are not, by themselves, more dangerous than progressing established tumors. Rather, new metastases are a marker of a loss of systemic disease control and reflect immunoevasion of occult micrometastatic sites. Therefore, patients with new metastases may benefit from an additional period of observation before intervention with invasive local therapy, in order to allow their disease biology to declare itself. In contrast, progression limited to established tumors leaves open the possibility that systemic disease control was achieved by effective immune clearance of clinically occult micrometastases, even if there are local failures. These patients seem most likely to benefit from local therapy, especially if they can be rendered NED. Thus, treatment failure after immunotherapy may be heterogeneous and require different management strategies.

Local therapy is extremely important for the management of CNS metastases and is supported by level 1 data [27–29]. We did not analyze intra-cranial procedures in this report but we did include some patients who were staged M1d before CPI who later required local therapy in extra-cranial sites (Table 1). All included patients staged M1d had brain metastases that either had been previously treated or had resolved with immunotherapy. Intra-cranial metastases are managed differently and may also be biologically unique; for example we showed that fewer patients with an ongoing complete response were staged M1d (Fig. 1). We did not attempt to explore the relationship between intracranial and extracranial metastases and our findings should not be extrapolated for the management of CNS metastases.

Local therapy, especially major surgery, can carry the risk of morbidity and be extremely costly for the health care system. Patients treated with local therapy in this study were highly selected. Most didn’t develop progression for more than a year after starting CPI, and many experienced impressive shrinkage or resolution of multiple metastases. Our hope is that the selection criteria we have described herein will help clinicians identify patients in whom aggressive local therapy is warranted, while simultaneously helping to spare from the morbidity of invasive procedures those who are unlikely to benefit. However, this study is greatly limited by its retrospective nature and the potential for selection bias. A prospective observational study at a minimum would be required to determine whether patterns of failure are useful for patient selection. Thus, our results should be interpreted with caution.

## Conclusions

Up to 18% of patients with oligoprogression after CPI may be candidates for local therapy, which can achieve durable PFS in selected patients. We have shown that long PFS is associated with progression limited to established tumors, while local therapy for new metastases is associated with early relapse and shorter survival. Local therapy to achieve NPRD may be useful to manage patients with discordant responses to CPI, although further study is needed. The curative potential of immunotherapy lies in its ability to completely eliminate both microscopic disease and macroscopic lesions. There may be a significant population of patients treated with CPI who experience microscopic clearance of systemic disease but have local immune failure in established lesions. These patients may be revealed by different patterns of failure after CPI and our experience shows that some of them can be cured by timely intervention with local therapy.

## Data Availability

All data generated or analyzed during this study are included in this published article and its supplementary information files.

## Abbreviations

ACT: Adoptive cell transfer
CPI: Checkpoint inhibitors
DSS: Disease-specific survival
NED: No evidence of disease
NPRD: Non-progressive residual disease
PFS: Progression-free survival
